# European Inflation in an American Mirror

**DOI:** 10.1007/s10272-022-1033-x

**Published:** 2022-04-08

**Authors:** Barry Eichengreen

**Affiliations:** Department of Economics, University of California, Berkeley, 30 Evans Hall, MC #3880, Berkeley, CA 94720-3880 USA

**Keywords:** E31, E52

## Abstract

In these highly uncertain times, flexibility has value.

Even before Russia's invasion of Ukraine, it had become painfully obvious that the United States had an inflation problem. Now Mr. Putin's war has added fuel to the fire by pushing up energy and food prices and creating additional supply-chain disruptions. It is clear that the Federal Reserve has fallen behind the curve, having failed to anticipate the magnitude of the inflation in the pipeline. What is not clear is whether and how it will now catch up. Nor is it clear whether other central banks, notably the European Central Bank (ECB), will avoid committing the same unforced error.

## Anchors aweigh

The numbers for the U.S. are not good. Annual inflation accelerated to 7.9% in February, the highest level since early 1982, propelled by rising prices of energy, shelter, food and motor vehicles. Alarmed observers point to parallels with the 1970s, when commodity prices shot up, the Fed fell behind the curve, and inflation expectations became unmoored. Consumers, producers and workers all expected prices to keep rising at the same or even at an accelerating pace. Accordingly, workers adjusted their wage demands, consumers their spending patterns, and businesses their prices, unleashing what became a self-fulfilling inflationary spiral.

The current situation is different. Inflation expectations, for the moment, remain at least tenuously anchored. The University of Michigan Survey of Consumers for February confirms that respondents expect inflation to approach 5% over the coming year, but then to fall back to just above 2% in the subsequent four years, consistent with the Fed's inflation target. The Federal Reserve Bank of New York's Survey of Consumer Expectations for the same month similarly shows that respondents expect inflation to run at 6% over the coming year but to then fall back to 3.8% over a three-year-ahead horizon. As of mid-March, the break-even inflation rate derived from five-year Treasury inflation-indexed securities shows expectations of inflation averaging around 3.5% over the next five years – meaning that inflation between 2023 and 2026 is expected to be somewhat lower, given expected inflation of 5% for 2022.[1] Although these numbers noticeably exceed the Fed's traditional 2% inflation target, it can be argued that they are not out of line with its new average inflation targeting framework, in which it seeks to keep inflation around 2% on average in the medium term, given how 2% was undershot in the preceding period.

The question, of course, is whether inflation expectations, however stable they are for the moment, will remain anchored in the future. Answering it, and assessing the analogy with the 1970s, requires understanding how those expectations became unmoored in that earlier instance. It requires determining whether the conditions leading to the "Great Inflation" have in fact been consigned to the dustbin of history. The answer, as with many things economic, turns out to be "yes and no."

## Dustbin of history

In 1973, consumer price inflation in the United States reached 6%, approaching where it is today. That inflation, like ours, was led by rising food and energy prices; then as now, there were sharp changes in relative prices below the surface of an accelerating headline inflation rate (Blinder, 1979).

Food and energy prices are volatile. This means that they can drop sharply, tamping down inflationary pressures, as well as continue to rise. The key question is thus why consumers, producers and workers, when forming expectations, extrapolated the elevated inflation rates of 1973-74 into the future. The answer is straightforward. The parties were more than justified for thinking that inflation would persist because there were absolutely no grounds for believing that the Federal Reserve would take action to tamp it down.

The Fed, or more specifically those responsible for its policies, were seen as unlikely to act because they lacked a coherent model of the connections between central bank policy and inflation. In the 1950s and first half of the 1960s, the anchor for policy, such as it was, was the Bretton Woods international monetary system. Under Bretton Woods, the U.S. pegged the dollar price of gold at $35 an ounce and stood ready to pay out gold for dollars on demand to foreign central

© The Author(s) 2022. Open Access: This article is distributed under the terms of the Creative Commons Attribution 4.0 International License (https://creativecommons.org/licenses/by/4.0/).

Open Access funding provided by ZBW – Leibniz Information Centre for Economics.

**Barry Eichengreen**, University of California, Berkeley, USA.

1 It can be argued (e.g. Christensen and Gillan, 2011) that the small size and limited liquidity of the Treasury Inflation-Protected Security market makes the implied breakeven rate an unreliable measure of expectations. This is, of course precisely the rationale for also considering other evidence such as survey data.

banks and governments.[2] The Fed understood that excessive inflation made possible by lax central bank policy might jeopardize this commitment. If U.S. interest rates were too low, capital would flow out of the country, gold would be lost to foreign entities acquiring dollars, and U.S. rates would have to be raised. If U.S. spending was too strong, imports would surge, gold would again be lost, and the Fed would be forced to rein in demand.

The Federal Reserve was not targeting inflation. It was not targeting high employment. Rather, it was seeking to preserve U.S. gold reserves and to defend the dollar's Bretton Woods peg. The minutes of the Federal Open Market Committee document these concerns (Bordo and Eichengreen, 2013). The value attached by the Fed to the stability of the exchange rate was public knowledge, by virtue of statements by members of the Board of Governors. Hence, if demand increased, fueling inflation and causing the balance of payments to deteriorate, there was an awareness that the Fed would tighten, which in turn limited the inflationary consequences. Expectations were anchored by the Fed's commitment to obeying what might be called the "Bretton Woods rules of the game."[3] This limited inflationary inertia and prevented inflation from taking off in response to shocks.

It is now commonplace to ascribe the advent of the Great Inflation to the 1971-73 collapse of Bretton Woods (Reis, 2021). In fact, however, Bretton Woods had already lost its bite, and inflation had already begun to accelerate, in the second half of the 1960s, prior to the 1971-73 Bretton Woods crisis.[4] In this earlier period, the U.S. adopted policies, such as an Interest Equalization Tax on American foreign financial investments, loosening the link between inflation and gold losses.[5] The Treasury Department asserted its responsibility for managing the foreign exchange market, allowing the Fed to dismiss gold losses and dollar weakness as someone else's problem. There was a decline after 1965 in the frequency of references in the minutes of the Federal Open Market Committee (FOMC) to exchange rate and balance of payments concerns (again see Bordo and Eichengreen, 2013).[6] As a result, U.S. inflation approached 6% already in 1970, even before the collapse of Bretton Woods.

Removing the exchange rate anchor would not have made a difference had the Fed possessed a coherent theory connecting monetary policy with inflation. Unfortunately, it did not. The closest thing to a theory was Chairman Arthur Burns' view that monetary policy did not matter. Burns saw inflation as caused, variously, by the excessive wage demands of unions, price increases by firms with market power, poor harvests and high oil prices, none of which monetary policy could directly control. He recognized a link between excessive budget deficits and inflation, but not one that a change in monetary policy could offset. In his academic career as a business cycle researcher, Burns had portrayed output and price fluctuations as shaped by institutional arrangements in product and labor markets, not by central bank policy (Burns and Mitchell, 1946). He brought to his tenure at the Federal Reserve this same focus on arrangements in labor, product, energy and commodity markets.

The next Fed chair, G. William Miller, lacked Burns' academic credentials and was not inclined to challenge the views of his illustrious predecessor. Eventually, Paul Volcker would have something to say about this, but not until the 1980s. Even then, it took Volcker time, and multiple tries, to alter expectations and firmly place the anchor.[7]

### The rocky road ahead

Circumstances today are different. Federal Reserve officials understand that, in all but the most exceptional circumstances, namely those of a liquidity trap, monetary policy and inflation are intertwined. They possess a coherent policy framework, namely average inflation targeting.

But circumstances today are also different from the 1980s, when Paul Volcker crushed inflation. The Powell Fed is strongly committed to not disturbing financial markets. It has communicated its intention of raising its policy interest rate in 25 basis point increments, presumably seven times between March and December of 2022, corresponding to its seven regularly scheduled Federal Open Market Committee meet-

2 This was in contrast to pre-1933 arrangements, when the authorities also committed to paying out gold at a fixed price, on demand, to private holders of dollars.

3 Barro (1982) suggests that what are referred to here as the Bretton Woods rules of the game were a continuation of the gold-standard rules of the game, and that the period after 1971 was "the first time that we [the United States] have completely severed, both currently and prospectively, the link between our money and a commodity base…"

4 ECB (2010) makes a similar point when comparing the U.S. and Europe in this period.

5 The Interest Equalization Tax was imposed in 1963 (Butterworth, 1970).

6 Specifically, we look at the Fed's policy actions between 1959 and 1971 and classify them according to whether or not they were predominantly motivated by balance of payments considerations, finding that such episodes were concentrated in the period ending in 1965.

7 In fact, contrary to legend, Volcker's early efforts to tame inflation were unsuccessful. Inflation expectations as measured by the Michigan survey continued to hover in the 9%-10% range following the October 6, 1979 press conference where Volcker announced the Fed's new operating strategy. When the Fed then paused its tightening cycle in late 1979 in response to a softening economy and fears of a recession, long-term interest rates rose, contrary to what one would expect given the impending recession and indicative of expectations of accelerating inflation. Progress on the inflation problem would have to wait. As late as the end of 1980, inflation expectations according to the Michigan survey were still as high as in late 1979, when Volcker's anti-inflation initiatives were just getting underway.

ings. It has so indicated through speeches by governors and Reserve Bank presidents and through FOMC statements and minutes. By relying so heavily on forward guidance, and by attaching such importance to the state of financial markets, it has effectively locked itself into that trajectory. Its fear is that moving faster, in response to more alarming inflation numbers, would constitute an unpleasant surprise for the markets. It might lead to a sharp correction in asset prices. A sharp shift in interest rates, by wrong-footing investment funds with leveraged positions in fixed-interest securities, might jeopardize financial stability. This is not the same as 1970s-style denial of the power of monetary policy. But it is evidence of a reluctance to use that power. Unfortunately, this nuance does not make the current policy stance less of a problem.

The issue is that seven 25 basis increases would leave the Federal funds target range at 1.75%-2% at the end of the year, and the real (inflation-adjusted) interest rate deep in negative territory. Federal Reserve policy would remain highly accommodating – in an economy with unemployment below 4% and inflation running well above target. For subduing inflation, the Fed would be relying entirely on declining spending, as the fiscal stimulus of 2021 recedes in the rearview mirror, and on increased supply, as global supply chains recover from COVID-19 era disruptions.

But, for better or worse, consumer spending shows little sign of declining. Although the federal government's stimulus checks may be an increasingly distant memory, households' excess savings, acquired in the pandemic period, still remain to be spent down. Now, moreover, there is the specter of new supply disruptions, as Chinese cities and factories lock down in response to the Omicron variant, containerships are caught in the Black Sea, and flows of energy, nickel and grain from Russia and Ukraine grow increasingly uncertain.

This means that the Fed should start laying the groundwork now for a series 50 basis point increases in rates in 2023. Recently, Chair Powell has taken a first step in this direction, but this remains only a first step. By following up with additional statements, the Fed can prepare the markets for the eventuality and avoid the financial volatility of which it is so fearful. It may then be able to move real interest rates back into positive territory by late 2023, when monetary policy will have become less accommodating. Even so, inflation is likely to remain notably above target for another year and a half, if not more.

## A premium on flexibility

A final important question is how different Europe is from the United States. The U.S. applied more fiscal stimulus, in general and specifically in 2021, creating more intense inflationary pressure. In addition, the "great resignation," as workers were detached from their employers during the pandemic, put more downward pressure on the U.S. labor supply, in contrast to Europe's more extensive policies designed to maintain those employment connections and to support continued labor-force participation (Pisani-Ferry, 2022). For both reasons, inflation prior to the eruption of war in Ukraine was more subdued in the euro area than the United States. Working in the other direction is that Europe is likely to see more inflation from rising energy prices, given that it is less self-sufficient in oil and gas. Inflation rose to 5.8% in February, on the back of rising food and fuel prices, lower than in the U.S. but still almost three times the ECB's inflation target. The central bank may be inclined to "look through" rising energy prices if their tendency to rise is only transitory. But it will not be able to look through recession risk if natural gas supplies from Russia are significantly curtailed, whether at the behest of President Putin or the West. By comparison, U.S. reliance on Russian oil and gas, and therefore the risk of a significant slowdown, are less.

Thus, while the risks in the U.S. are clearly tilted toward inflation, those in the euro area are more evenly balanced. Fortunately, the ECB does not share the Fed's fixation on the reaction of financial markets, perhaps because Europe has a more heavily bank-based and less market-based financial system. The ECB provides forward guidance, but by the standards of the Fed it is relatively vague. Some observers criticize President Lagarde and the ECB for unclear communication. But the positive interpretation is that the ECB has not gone as far as the Fed in locking itself into a policy position for 2022. This is good. If conditions call for it to accelerate the normalization of policy rates, the ECB will be in a better position to act. Equally, if events in Russia and Ukraine instead lead to an economic slowdown in Western Europe, it will be in a position to pivot. In these highly uncertain times, flexibility has value.
